# MYC chromosomal aberration in differential diagnosis between Burkitt and other aggressive lymphomas

**DOI:** 10.1186/1750-9378-8-37

**Published:** 2013-09-30

**Authors:** Gabriella Aquino, Laura Marra, Monica Cantile, Annarosaria De Chiara, Giuseppina Liguori, Maria Pia Curcio, Rocco Sabatino, Giuseppe Pannone, Antonio Pinto, Gerardo Botti, Renato Franco

**Affiliations:** 1 Pathology Unit, "Istituto Nazionale Tumori Fondazione G. Pascale" - Irccs, Naples, Italy; 2 Medicine and Surgery Department, Foggia University, Foggia, Italy; 3 Haematology Unit, "Istituto Nazionale Tumori Fondazione G. Pascale" - Irccs, Naples, Italy

**Keywords:** Burkitt Lymphoma, FISH, MYC, Aggressive non-Hodgkin B-cell lymphoma, Diffuse large B cell lymphoma, B-cell lymphoma unclassifiable

## Abstract

*Myc* oncogenetic deregulation is abundantly described in several solid human cancer and lymphomas. Particularly, Burkitt's lymphoma belongs to the family of B Non Hodgkin aggressive lymphomas. Although it is morphologically characterized, immunophenotypic and cytogenetic diagnosis remains complex. In 2008, the WHO has introduced a new diagnostic class of aggressive B-cell lymphomas with features intermediate between BL and DLBCL. This diagnostic class represents a temporary container of aggressive B-cell lymphomas, not completely belonging to the BL and DLBCL categories. The importance of establishing a correct diagnosis would allow a better prognostic classification and a better therapeutic approach. In this review, we summarize the main diagnostic approaches necessary for appropriate diagnoses and we emphasize the importance of cytogenetic analysis of the oncogene *Myc* in the histopathological diagnosis and the prognostic/predictive stratification. In this contest, *Myc* represents the more involved gene in the development of these lymphomas. Therefore, we analyze the genetic aberrations causing its over-expression and the concomitant deregulation of molecular pathways related to it. We also propose a FISH approach useful in the diagnosis of these lymphomas.

## Introduction

Chromosomal translocations involving the immunoglobulin genes are common in B-cell non-Hodgkin lymphomas [1,2]. Some translocations are characterizing specific lymphoma histotypes and are often considered as cancer-initiating events [3]. For instance, t(8;14)(q24;q32), that involves *Myc* and IgH genes, is generally considered a hallmark of Burkitt Lymphoma (BL), but this translocation is not the only cytogenetic alteration observed in this type of lymphoma. BL is an aggressive non-Hodgkin B-cell lymphoma (B-NLH) characterized by the most rapidly growing cells [4]. It represents the first human tumor associated to a specific viral infection and one of the first with a chromosomal rearrangement activating an oncogene [5,6]. Recent evidence suggests that lipid pathway is altered in BL. Indeed neoplastic cells are characterizated by the accumulation of lipid vacuoles [7]. Conventionally three clinical variants of BL have been described: endemic (eBL), sporadic (sBL) and HIV–related [8]. Histologically BL shows a "starry sky" appearance, due to death cells and scattered tingible-body-laden macrophages present in monomorphic B-cell population background and a high proliferation rate is always demonstrated [4]. Although these morphological characteristics are observed in the BL, in adults a reliable diagnosis is very difficult to produce, since a subset of lymphomas with morphological features similar to BL are described [9]. Particularly differential diagnosis from some cases of diffuse large B cell lymphoma (DLBCL) and from B-cell lymphoma, unclassifiable, often results difficult. Even with the use of current diagnostic criteria, the distinction is not precise; in fact the agreement among expert hematopathologists on the pathological diagnosis of this subset of aggressive B lymphomas is only 53 percent [10,11]. The distinction between BL and DLBCL is clinically important, because these lymphomas are treated with different chemotherapeutic protocols and differ in their outcome [12].

Adult BL shows a rapdly developing disease, so diagnosis and staging are urgent because aggressive high-dose chemo-therapy should be started as soon as possible. Aggressive prophylaxis must be started immediately after diagnosis is confirmed [13]. However the interpretation of response is difficult because there isn’t a single protocol [14]. In addition recently Rituximab has also been introduced for treatment of BL and B aggressive lymphomas [15].

## Review

### MYC physiology

**Figure 1:**
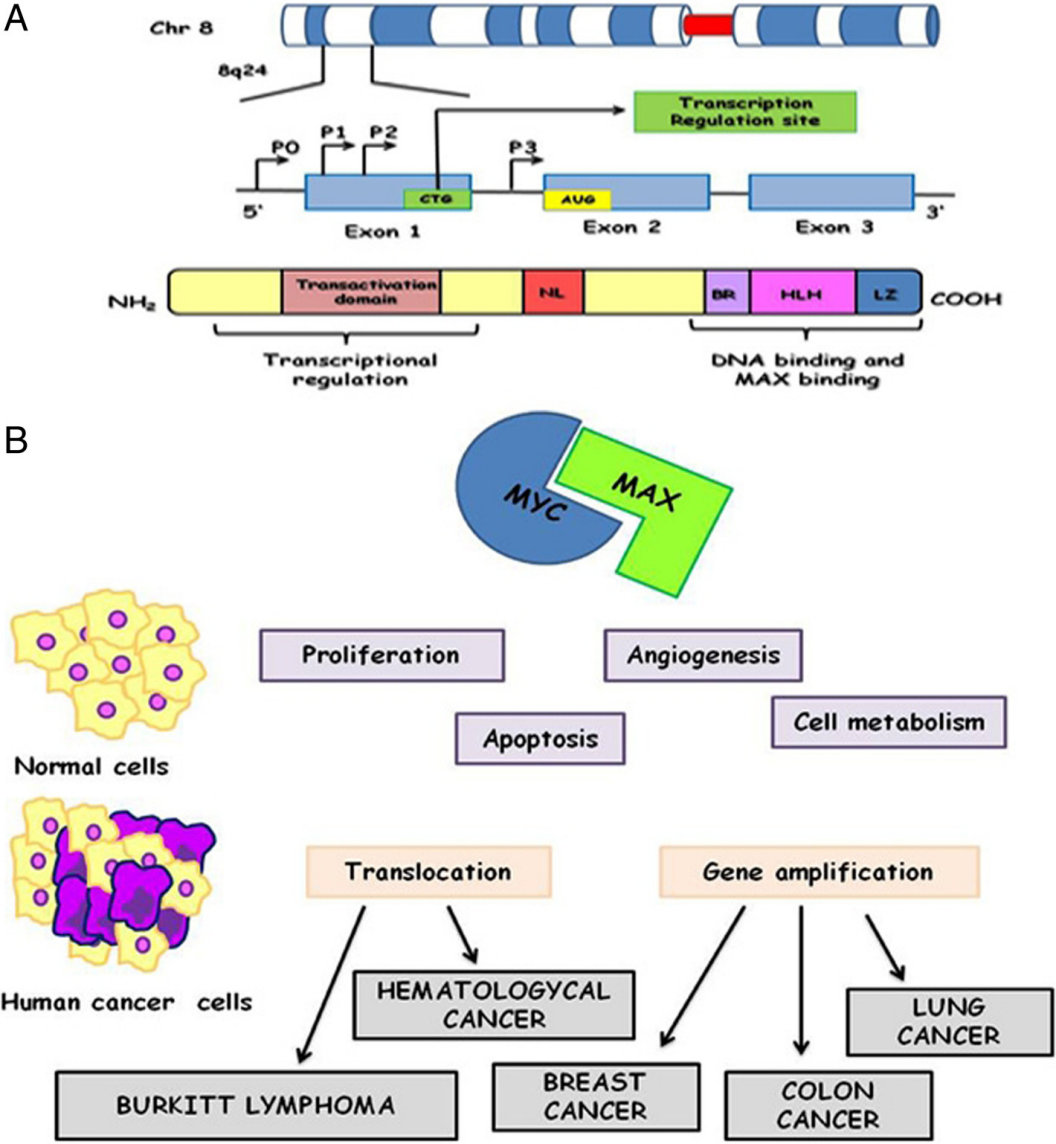
**MYC. A)** Schematic representation of MYC gene and protein domains; **B)** MYC alteration in human cancer.

C-Myc is a transcription factor, playing a role in the control of the cell cycle progression. *C-Myc* belongs to a transcription factors family that includes MYCL (L-Myc) and MYCN (N-Myc) and it is located on 8q24 chromosome [16]. *Myc* gene is composed by three exons and Myc mRNAs generate two isoforms of Myc polypeptides: the first, the shorter one, starts an internal AUG, the second, that is longer, initiates at a CUG [17]. The shorter one plays an important role in the stress response [18,19] (Figure 1). In addition, it is involved in the regulation of many other biological activities, such as differentiation, apoptosis, angiogenesis, cell adhesion and motility, telomerase activity and cell metabolism [20]. *Myc* is considered the human oncogene more frequently deregulated in many types of cancer with subsequent, uncontrolled cell proliferation, genomic instability, apoptosis, escape of immune surveillance and cell immortalization [21,22].

Myc protein contains a basic region helix-loop-helix-leucine zipper and modulates the expression of target genes by binding to specific DNA sequences (E-Box). Myc performs its function by dimerization with MAX, a leucine-zipper transcription factors family. Recently, it has been demonstrated that c-*Myc* expression in GCs (Germinal Center) is lower compared to naive and memory cells. Probably, this low expression could protect against Myc-induced genomic instability in the GC [23].

### Myc alteration in human cancer

*Myc* oncogenetic deregulation could be induced by point mutations, gene amplification, translocation, epigenetic reprogramming, enhanced translation and increased protein stability [21]. The effects are C-Myc protein overexpression, demonstrated in 80% of breast cancers, 70% of colon cancers, 90% of gynecological cancers, 50% of hepatocellular carcinomas, 30% of lung cancer and a variety of hematological tumors [24]. Aberrant *Myc* expression has also been identified in Prostatic Cancer where it has been proposed as a potential prognostic factor [25,26] (Figure 1B). Generally *Myc* gene amplification has been described as the most frequent molecular alteration in most of solid tumours [21]. In addiction Single-nucleotide Polymorphisms (SNPs) within 8q24 chromosomal region have been found to be associated with colorectal, breast, bladder, ovarian and prostate cancers [26,27]. Furthermore, point mutations are revealed in Myc N-terminal domain (residues 44-65), in particular, the most frequently mutated residue is Thr-58. The phosphorylation of this residue has been shown to control c-Myc degradation and mutations, abolishing Thr-58, lead to an increased c-Myc half life in BL [28,29]. The translocation t(8;14) has been described as the most frequent aberration involving *Myc* gene in BL with the immunoglobulin heavy chain (IgH) gene as partner. Less common aberration involves light chain immunoglobulin genes (Igλ or Igκ) in the translocations t(2;8) and t(8;22) [30]. The activation of the *Myc* gene at 8q24 is considered the main pathogenetic feature of BL, but the contribution of other genetic mutations to the disease is an important developing point [30]. In addition *Myc* translocation is not only specifically observed in BL but it can occur in other hematological malignancies. Indeed *Myc* rearrangement is observed in 5-10% of diffuse large B-cell lymphomas and up to 50% of high-grade B-cell lymphomas other than Burkitt lymphomas [31]. In these tumours, *Myc* translocations can also involve non-*IG* partners [24].

### Molecular pathways associated to MYC overexpression in Burkitt lymphoma

*Myc* translocation in BL is considered as a lymphoma initiating event, in other lymphomas it may also occur as a secondary event during disease progression [3]. Well documented oncogenetic alterations are associated to other intracellular pathways.

Recently C. Love et al. have highlighted a series of gene mutations in BL. For example, ID3 (inhibitor of DNA binding protein) gene mutations produce a twofold higher gene expression in BL cells when compared to DLBCL [32]. ID3 mutation is associated to increased G to S phase cell cycle progression correlated to higher expression of cell cycle pathway genes, such as E2F1, CDK7, MCM10 and with an higher expression of known Myc target genes. This phenomenon, through ID3 mimetics, could represent the possibility of a potential therapeutic approach in BL [32].

Using animal models, many studies have shown that the translocation involving *Myc* could be mediated by citidinedeaminase (AICDA) and that it is not activated by the recombinase (RAG1/2). This suggests the presence of somatic hypermutation or class switch recombination, that may be detected in normal tissue [33,34].

Several papers described an overexpression of NF-kB (Nuclear Factor kappa-light-chain-enhancer of activated B cells) both in DLBCL and HL and its lower expression in BL. Klapproth et al. suggest in a study realized on mice and human that in *c-Myc* transformated lymphoma cells, NF-kB-pathway is deregulated. Therefore they concluded that c-MYC overexpression sensitizes cells to NF-κB–induced apoptosis, and the absence of NF-κB signaling is an assumption for MYC-mediated tumorigenesis [35].

Another study tried to explain the possible role of IP3K (Inositol 1,4,5-Trisphosphate 3-kinase) and *Myc* in primary events of lymphomagenesis, using mouse models of BL. The results show a significant activation of IP3K pathway, especially in cells where the signal of NF-kB is off [36]. Recently, IP3K overexpression has been found in human BL, suggesting a functional role in BL pathogenesis [35]. The involvement of NF-kB and IP3K pathways might have implication for the development of therapies against MYC-positive tumours. Recent studies highlight c-*Myc* influence on the Retinoblastoma (Rb) pathways. Rb gene family is composed by three (Rb, pRb2/p130 and p107) cell cycle regulator protein members. Cinti et al. showed that several genetic alterations disrupt the nuclear localization of the retinoblastoma-related gene RB2/p130 in human tumor cell lines and primary tumors [37]. In particular, mutation of *RB2/p130* caused the upregulation of cyclins E1 and A2, involved in cell cycle progression from G1 to S-phase, and the inactivation of the transcription factor E2F4 (typically increasing during the S phase) [38]. This alteration is more frequent in endemic BL and lesser in sporadic BL. However, *RB2/p130* mutation has not been included into molecular signatures that distinguish BL and DLBCL, suggesting that RB2/p130 deregulation was common in all B–non-Hodgkin lymphomas (NHLs), and not exclusive for BL pathogenesis [39].

**Figure 2:**
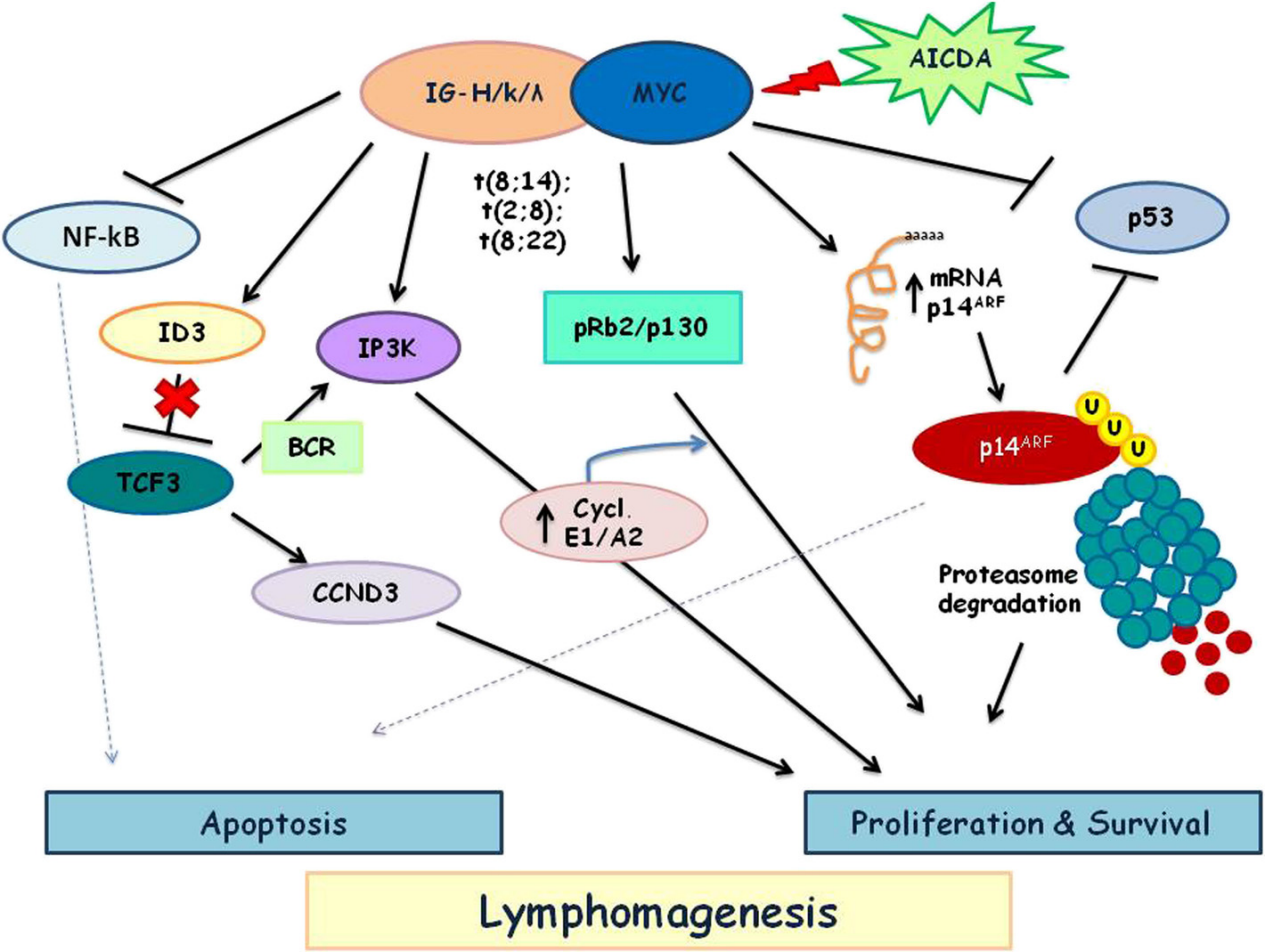
Schematic representation of MYC translocation and molecular pathways involved in lymphomagenesis.

The INK4/ARF locus encodes two tumour suppressor genes, p16 and p14 that distinctively regulate Rb and p53 pathways. Human and murine studies have showed that the simultaneous c-Myc iperactivation and INK4/ARF inactivation are an essential step during the development of BL, conferring a further growth advantage and apoptosis protection to the cells [40,41]. Moreover, c-Myc, p14 and p16 are degraded with proteasome-dependent mechanism, and a less ubiquitination is demonstrated in BL. These evidences suggest that proteasome inhibitors may be further considered in the treatment of BL [42]. Recent studies have highlighted that miRNAs (small non coding RNA) may have a role in malignant transformation in several solid tumors, but little is known about their expression and deregulation in malignant lymphomas [43]. In particular, hsa-miR-155 was found to be highly expressed in 90% of Hodgkin's lymphomas and in diffuse large B-cell lymphomas. Moreover recent studies have underlined its role in B-cell differentiation. Furthermore, the miR-17-92 cluster was described as a transcriptional target of c-Myc and it was over-expressed during lymphomagenesis [44]. Other studies have assessed that hsa-miR-127 up-regulation in EBV positive BL confirming different pathogenetic mechanisms between EBV-positive and EBV-negative BL [45]. We have schematized in Figure 2 all the previously discussed pathways correlated to *Myc* gene aberration.

### Detection of *MYC* gene/protein alteration

Several studies have demonstrated distinctive complex karyotypes (CK) in BL and DLBCL. Havelange et al. have identified recurrent alterations associated with *Myc* rearrangements in 84 aggressive B-cell lymphomas by multicolor fluorescence in situ hybridization (M-FISH). They concluded that BL karyotypes were less complex and aneuploid than other lymphomas with *Myc*-rearrangement. This condition suggests that BL with CK are indeed different from other aggressive MYC-rearranged lymphomas, usually showing wider genetic complexity [46]. Several Comparative genomic hybridization (CGH) studies highlight genomic imbalances in BL. Garcìa et al. found chromosomes 12q, Xq, 22q, 20q, 9q gains, and chromosomes 13q and 4q losses. Moreover they found high level of amplification in the regions 1q23-31, 6p12-p25, 8p22-23 [47]. Anyway previous studies have determined that BL has a simple karyotype < or=2 additional abnormalities and it is associated to better overall survival respect to other B aggressive lymphomas, this condiction is generally observed in BLs [48]. Gains or amplifications of chromosomes 1q and 7q (respectively 20% and 10% of BL) have been associated with worse clinic outcome and also all 13q chromosomal abnormalities have been related to an aggressive behaviour [47,49,50]. Furthermore, recently, a comparative analysis between whole-genome oligonucleotide array CGH analysis and FISH in a Burkitt's lymphoma-derived cell lines, showed three minimal critical regions (MCR) localized on Chr 1q harboring several genes such as BCA2, PIAS3 and MDM4 and AKT3. These regions appear critically involved in BL prognosis [51].

However, the *Myc* translocation remains the main cytogenetic signature of BL as shown by its routinely use in several diagnostic algorithms. This investigation is fundamental in differential diagnosis with other lymphomas morphologically similar to BL but with atypical immunophenotype or genetic signatures.

Hummel et al. proposed a "BL similarity index", based on the analysis of 58 genes, classifying aggressive NHL into molecular BL (mBL), intermediate cases, and non molecular Burkitt. They analysed 220 mature aggressive B-cell lymphomas, and identified a consistent gene panel characteristic of molecular BL. These genes also included several target genes of the nuclear factor-κB pathway (i.e., *BCL2A1, FLIP, CD44, NFKBIA, BCL3,* and *STAT3*) that normally distinguished activated B-cell-like (ABC) or germinal-center B-cell-like lymphomas (GC) [52,53]. Through this index yet not all cases with morphologic or immunophenotypical features of Burkitt’s lymphoma were classified as mBL. Molecular signature was strongly supported from the genetic analysis that defines three groups: i) the myc-simple group characterized by *IG-Myc* fusion and a low number of chromosomal imbalances (complexity score < 6, *MYC* translocation could be the primary oncogenic event) largely overlapped with the molecular BL and associated with a favourable clinical outcome; ii) *Myc*-complex status (complexity score > 6, *Myc* translocation could be the second oncogenic event) associated with a poor outcome, independently of age and clinical stage corresponding to the intermediate group; iii) *Myc* negative group including non molecular Burkitt cases [53].

On the basis of current literature, Bellan et al. suggested a practical approach for BL diagnosis. To distinguish among BL, DLBCL and the provisional category of "unclassifiable B-cell lymphoma", with intermediate features between DLBCL and BL (BCLU), they used cytogenetic, molecular and immunohistochemical techniques, selecting a large panel of antibodies. In particular, FISH analysis was performed to detect the translocations involving *Myc*, *BCL2* and *BCL6* through commercially available probes [54].

**Figure 3:**
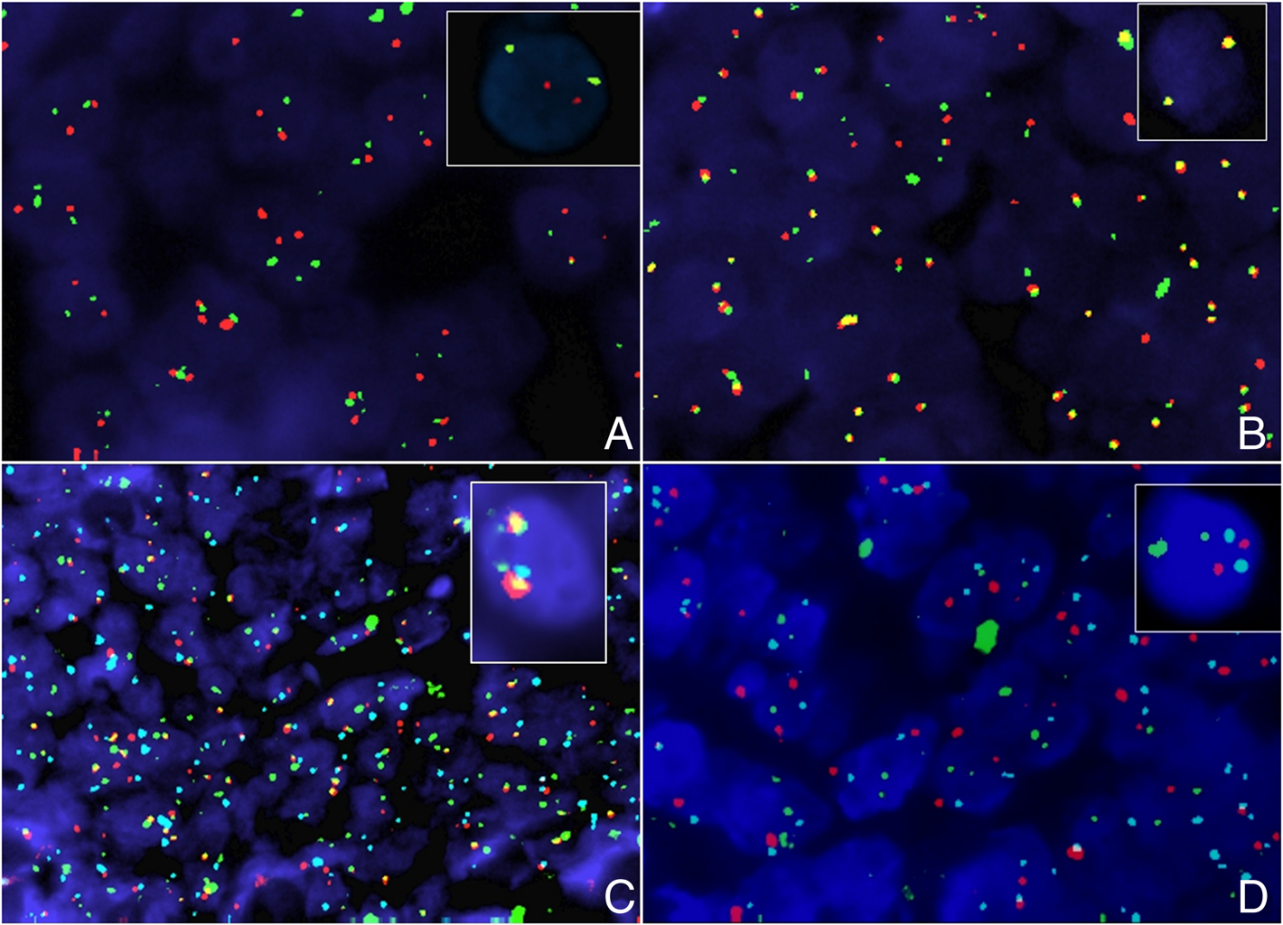
**Above, FISH assay shows the MYC locus rearrangement with a break-apart probe (Vysis LSI MYC Dual Color Break Apart Rearrangement Probe Kit). A**. MYC rearrangement-positive tumor cells, showing one yellow fusion signal, one orange and one green signals; **B**. Absence of MYC translocation, showing fusion signal patterns; Below, FISH assay shows the IGH/MYC/CEP8 Tri-Color Dual Fusion probe (Vysis IGH/MYC/CEP 8 Tri-Color DF FISH Probe Kit): **C**. The reciprocal t(8;14) in tumor cells showed a pattern of one orange, one green, two orange/green fusions, and two aqua centromeric signals; **D**. Absence of reciprocal translocation showed the two aqua, two orange, and two green signals pattern.

*Myc* translocation could be easily detected through the use of commercial probes by breaking apart or by dual fusion strategy used for FISH analyses. The first strategy allows, in a single approach, to assess the integrity of the *Myc* gene, but it does not provide information about the translocation partner. Known translocation partners of *Myc* gene could be detected using the second strategy (Figure 3). Several commercial probes are available, but they detect separately the different *Myc* partners. In fact some Break Apart probes, investigating the presence of *Myc* rearrangements in the locus corresponding to Igλ or Igκ, are available. The only commercial dual colour dual fusion *Myc* probe allows the translocation t (8:14) assessment. There are no commercial probes to investigate the *non-IG Myc* partners. The *Myc* break apart probe combined with the previously described probes are necessary to detect this subset of translocations.

Naresh et al. proposed in the diagnostic approach of BL an immunohistochemistry and FISH scoring system, based on three phases. Particularly, the first phase is based on the scoring of both the morphological features and a small immunohistochemistry panel detections, including BCL2 and CD10 antibodies. If the cumulative score is ≥ 5, the diagnosis of BL can be proposed; if the score is < 3 the diagnosis is not BL. The unresolved cases with intermediate score should be further scored through a larger panel of immunohistochemistry, such as ki67 (score 0-1-2), CD38 (score 0-1) and CD44 (score 0-1). Only if cumulative scores is ≥8, diagnosis of BL is definitively proposed. Finally, If this phase is uncertain, FISH analysis, including Myc\IG translocation and rearrangements of BCL2 and BCL6 should be crucial. If cumulative scores is ≥8: diagnosis of BL; if the score is between 6 and 7: BL not excluded. Through this approach is possible enable to give lead to a precise diagnosis of BL in more than 90% cases [55].

Instead, Salaverria et al. propose a genetic model of pathogenesis of high-grade B cell lymphomas "gray zone", related to genetic aberration and age. The gray zone represents hybrid zone between classical BL and classical DLBCL, and contain "secondary Burkitt-transformed lymphomas" and many indeterminate lymphomas. Cytogenetically Sporadic BLs in children and adults are similar. Both groups are characterized by the same low genomic complexity including the same genetic aberrations. A correct subclassification of mature aggressive B-cell lymphomas in adults directly influence the therapeutic strategy. This genetic model shows that real adult BLs are very rare and that the BL with more genetic alteration is extremely difficult to find. In this contest, the distinctive feature of BL is represented by its low genomic complexity [56].

*Myc* translocation has not only diagnostic value but it is also a powerful prognostic indicator in several lymphomas.

*Myc* translocation is associated to poor prognosis in other B aggressive lymphomas. Indeed LI S. et al. showed several lymphoma cases with a germinal center B-cell immunophenotype carrying *Myc* and BCL2 rearrangements and clinically aggressive behaviour, independently of their morphological appearance [57]. Pei Lin et al. assert that the only *Myc* aberration, in unclassifiable B-cell lymphoma, identifies patient subsets, requiring more aggressive therapy than R-CHOP [58].

However, *Myc* can be regulated by other mechanisms inducing its increased protein expression and its hyperactivation [59,60]. FISH is unable to detect genetic deregulation that affects gene expression on the transcriptional and translational levels unlike immunohistochemical analysis [61]. For a long time the Myc evaluation by immunohistochemestry has been hampered by a lack of anti-MYC antibodies that are suitable to detect the increased protein expression. However, recently a new commercial, Myc antibody (clone Y69; Epitomics, Burlingame, CA) seems to be useful in the diction of myc overexpression, independently from molecular mechanism. This monoclonal antibody targets the Myc protein-N terminus. This Myc antibody shows a typical nuclear staining and it has been proposed a significant diagnostic cut-off in BL for the immunoscoring when higher than 40%. Several recent studies proposed to introduce this antibody in a novel diagnostic alghorithm.

Green et al. analyse a group of DLBCL both trough immunohistochemistry for Myc, BCL2, CD10, BCL6, and MUM1/interferon regulatory factor 4, and FISH for *Myc* and BCL2. They concluded that FISH analysis identified Double-Hit Lymphoma (DHL) in 6% of patients, while immunohistochemical MYC and BCL2 analyses identified a double-hit group that comprised 29% of patients. These cases were significantly associated with shorter OS (P < .001), and shorter progression-free survival (PFS; P < .001), concluding that the only MYC and Bcl2 immunohistochemestry defined a large subset of DLBCLs strongly associated with poor outcome in patients treated with R-CHOP [62].

Finally, Horn et al. proposed to introduce a novel diagnostic approach using Myc antibody. They suggest a combined immunohistochemical and FISH score to predict outcome in DLBCL patients (score 0, when BCL2 <70% and MYC <40%, score 1 MYC and Bcl2 expression near to cut off and score 3, when Bcl2 and MYC expression is more than cut-off) [63].

The routinely diagnostic application of Myc antibody is not yet applied. Recent studies show Myc antibody use in DLBCL where the percentage of *Myc* translocation is very low [64]. In the future Myc antibody could be used for the other B aggressive lymphomas, BL and BCLU, to detect Myc altered expression independently from the mutations.

## Conclusion

### Diagnostic algorithms

Detection of the *Myc* translocation is currently performed by conventional cytogenetics, Southern blot, and polymerase chain reaction–based methods. Nevertheless, all these methods can fail to detect *IG-M*yc fusions [65]. The most reliable method is cytogenetic analysis by fluorescence in situ hybridization (FISH). Nowadays the molecular genetics is fully integrated into the routine diagnostic of lymphomas. The Gold standard method is the CGH Array but this analysis has not been introduced into routine diagnostic laboratories because it is labour-intensive and it has a high rate of failure [29]. Thus in the future, the development of a FISH assay for simultaneous detection of all known Burkitt abnormalities will be necessary.

**Figure 4:**
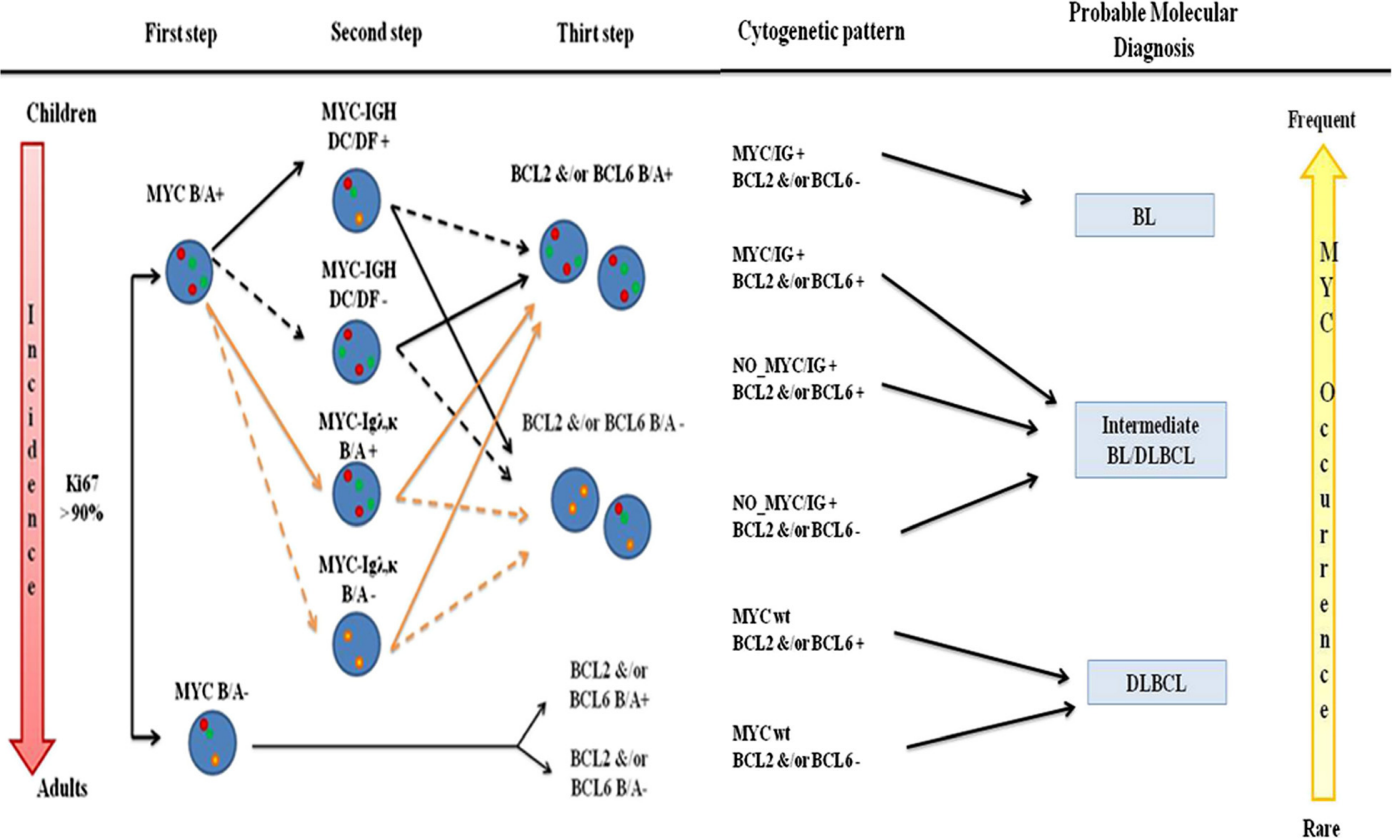
**The cytogenetics diagnostic algorithm.** MYC rearrangement is recommended on lymphoma cases with increased Ki67 expression (> 90%). Molecular Burkitt Lymphoma (BL) is defined only by the presence of the MYC-IG rearrangement, an early and frequent event; Diffuse Large B-cell Lymphoma (DLBCL) is cytogenetically set by the absence of MYC aberration; Intermediate BL/DLBCL are defined by unstable phenotype, generally MYC rearrangement is a second event and it doesn’t involve IG locus.

We summarized a cytogenetic diagnostic "flowchart" for a better and safer histopathologic diagnosis of Burkitt (Figure 4). We recommend a first FISH approach using *Myc* Break Apart probe on lymphoma cases with increased (>90%) Ki67, to identify all positive samples for *Myc* translocations. BCL2 and BCL6 translocations using Break Apart probes will be performed on all negative samples. Finally on the positive specimens should evaluate also the presence of *IG-Myc* translocation through the use of a Dual colour dual fusion *Myc-IGH* probe and *IGK* and *IGL* Break Apart probes. Then, *BCL2* and *BCL6* status should be investigated. In our opinion, this is the best approach to avoid misdiagnosis of molecular BL but it is useful only if it is integrated with morphologic and immunophenotypic evaluation. The diagnosis of BL and other aggressive B cell Lymphomas, with or without *Myc* breakpoints, represents an important start-point for future clinical trials to establish different therapeutical strategies for these lymphomas. Although the FISH-based algorithmic approach results an important tool for BL diagnosis, it is not easily accessible in most of the pathology laboratories because it is an expensive method and so it remains a speculative analysis.

## Competing interests

The authors declare that they have no competing interests.

## Authors’ contributions

GA and LM drafted the manuscript. All authors read and approved the final manuscript.
